# Evaluation of Vincamine Loaded with Silver Nanoparticles as a New Potential Therapeutic Agent Against Ehrlich’s Solid Carcinoma in Mice

**DOI:** 10.3390/cells13211762

**Published:** 2024-10-24

**Authors:** Naief Dahran, Mohamed S. Othman, Mohamed E. Ghoniem, Mai A. Samak, Mohamed T. Elabbasy, Sofian T. Obeidat, Ghada M. Aleid, Shimaa Abo Elnaga, Azza M. Khaled, Aya A. Altaleb, Ahmed E. Abdel Moneim

**Affiliations:** 1 Department of Basic Medical Sciences, College of Medicine, University of Jeddah, Jeddah 21959, Saudi Arabia; ndahran@uj.edu.sa; 2 Biochemistry Department, College of Medicine, University of Ha’il, Ha′il 2440, Saudi Arabia; g.aleid@uoh.edu.sa (G.M.A.); a.khaled@uoh.edu.sa (A.M.K.); 3 Faculty of Biotechnology, October University for Modern Science and Arts (MSA), Giza 12566, Egypt; 4 Department of Internal Medicine, College of Medicine, University of Ha’il, Ha’il 2240, Saudi Arabia; mo.ghonim@uoh.edu.sa; 5 Department of Internal Medicine, Faculty of Medicine, Zagazig University, Zagazig 44519, Egypt; 6 College of Medicine, University of Ha’il, Ha’il 2240, Saudi Arabia; dr.maiamin@yahoo.com (M.A.S.); tharwat330@gmail.com (M.T.E.); 7 Department of Medical Histology and Cell Biology, Faculty of Medicine, Zagazig University, Zagazig 44519, Egypt; 8 Basic Sciences Department, Deanship of Preparatory Year, University of Ha’il, Ha′il 2440, Saudi Arabia; s.obeidat@uoh.edu.sa (S.T.O.); shimaasmart@yahoo.com (S.A.E.); 9 College of Medicine, University of Gazi, Ankara 06500, Turkey; aya.altaleb@gazi.edu.tr; 10 Zoology and Entomology Department, Faculty of Science, Helwan University, Ain Helwan, Cairo 11795, Egypt; 11 Al-Ayen Scientific Research Center, Al-Ayen Iraqi University, AUIQ, An Nasiriyah, Thi Qar P.O. Box 64004, Iraq

**Keywords:** vincamine, Ehrlich solid carcinoma, apoptosis, anticancer, angiogenesis

## Abstract

Vincamine, a monoterpenoid indole alkaloid with vasodilatory properties, is extracted from the leaves of *Vinca minor*. The present study aimed to determine the potential anticancer effects of vincamine loaded in silver nanoparticles (VCN-AgNPs) in mice with Ehrlich solid carcinoma (ESC). After tumor transplantation, the mice were divided into five groups: ESC, ESC+Cisplatin (CPN; 5 mg/kg), ESC+VCN (40 mg/kg), ESC+AgNPs (6 mg/kg), and ESC+VCN-AgNPs (20 mg/kg). The administration of VCN-AgNPs to ESC-bearing mice improved their survival rate and reduced their body weight, tumor size, and tumor weight compared to the ESC group. Furthermore, VCN-AgNPs intensified oxidative stress in tumor tissues, as evidenced by elevated levels of lipid peroxidation (LPO) and nitric oxide (NO), along with a reduction in the levels of the antioxidants investigated (GSH, GPx, GR, SOD, CAT, and TAC). Furthermore, VCN-AgNPs increased the apoptotic proteins Bax and caspase-3, decreased the anti-apoptotic protein (Bcl-2), increased the inflammatory markers TNF-α and IL-1β, and inhibited angiogenesis by lowering VEGF levels in tumor tissues, all of which led to apoptosis. Furthermore, histopathological studies showed that VCN-AgNPs suppressed the progression of Ehrlich carcinoma and induced the formation of clusters of necrotic and fragmented tumor cells. VCN-AgNPs possess cytotoxic and genotoxic effects against ESC because of their pro-oxidant, pro-apoptotic, pro-inflammatory, and antiangiogenic effects. Additionally, the combination of VCN-AgNPs was more effective and safer than chemically synthesized AgNPs, as indicated by an increase in the lifespan of animals and the total tumor inhibition index.

## 1. Introduction

Cancer is a progressive disease and one of the most important causes of death worldwide. In the last century, in the majority of nations, cancer has surpassed cardiovascular diseases as the primary cause of premature mortality due to the current increasing trends in the main cancer types. In 2020, more than half of all cancer deaths were premature, accounting for 182.8 million life years lost to illness globally [1].

Worldwide, there is significant investment in cancer prevention, diagnosis, and treatment. Chemotherapy is one of the most sought-after treatments for cancer. Nevertheless, chemotherapy’s downfall is that it also attacks normal healthy cells, leading to severe and toxic side effects, e.g., vomiting, anemia, fatigue, diarrhea, hair loss, nausea, and damage to the immune system. Furthermore, the majority of anticancer drugs have poor selectivity and action against tumors, and the most prevalent malignancies are now growing more resistant to the anticancer medications that are now on the market [2]. It is obvious that there is an urgent need for the development of innovative treatments that are more effective while having fewer negative side effects.

Recently, a significant amount of research has been conducted on plant-derived medicinal substances as potential sources for developing anticancer medications with fewer negative side effects [3]. Almost two-thirds of FDA-approved cancer medications have been inspired by or drawn from natural sources [4,5].

Pharmacological investigations of the alkaloids of minor plants of *Vinca* and their derivatives, such as vincristine, vinorelbine, vinblastine, and vindesine, proved their anticancer properties. Approximately 25 to 65% of the indole alkaloids in *Vinca minor* are vincamine (VCN), which has the molecular formula C_21_H_26_N_2_O_3_. It is frequently used for diseases of the circulatory system, cerebrovascular diseases, psychological productivity, and poor concentration, as it has been proven to help brain metabolism, increase oxygen supply, prevent brain cell ageing, and improve memory and cognitive ability [6]. Few recent studies have attempted to study the anticancer effect of VCN in cancer cell lines in vitro, but the limited solubility and bioavailability of VCN may limit its anticancer effect.

The utilization of nanotechnology in combination therapy has emerged as an encouraging direction for cancer treatment as a result of its enhanced effectiveness and reduced occurrence of unwanted effects. The abilities of nanomaterials to carry thousands of drug molecules while overcoming problems with solubility, stability, and resistance, as well as the potential for targeted delivery to the tumor, are the main benefits of using them as carriers for anticancer agents [2]. The easy surface modification and synthesis of silver nanoparticles, strong increases, and exceptional biocompatibility have drawn a great deal of interest in cancer research [7].

To our knowledge, no studies have investigated vincamine’s in vivo antineoplastic activity. Furthermore, no studies have explored the antitumor activity of vincamine-loaded silver nanoparticles. The goal of this study is to evaluate the potential antiproliferative effect of silver nanoparticles loaded with vincamine in the treatment of Ehrlich solid carcinoma in mice. Various biochemical, histological, immunohistochemical, and molecular approaches are used to assess the anticancer and antimetastatic activities.

## 2. Materials and Methods

### 2.1. Drugs and Chemicals

Sigma-Aldrich (St. Louis, MO, USA) supplied cisplatin (CPN) and vincamine (VCN). Cisplatin was dissolved in saline at a concentration of 1 mg/mL. Vincamine was suspended in 1% Tween 80 at a concentration of 10 mg/mL. All reagents used were of analytical grade.

### 2.2. Chemical Synthesis of Silver Nanoparticles

Silver nanoparticles were prepared as described by Dong et al. [8]. Briefly, 29% AgNO_3_ in H_2_O was stirred over a hot plate, and trisodium citrate (0.1 M) was added dropwise until the color deepened.

### 2.3. Biogenic Synthesis of VCN-AgNPs

Green synthesis of AgNPs was achieved by adapting a modified version of the approach developed by El-Khadragy et al. [9]. Concisely, a 5 mL solution of VCN with a concentration of 0.1 mM/mL was slowly added to a 5 mL solution of AgNO_3_ with the same concentration. The mixture was then agitated at a temperature of 50 °C for 1 h. Additionally, the average dimensions of the AgNPs and VCN-AgNPs were determined using a Zetasizer (ZEN 3600, Malvern Instruments, Malvern, UK), while the size and morphology of the nanoparticles were examined using transmission electron microscopy (JEM-1230, JEOL Ltd., Peabody, MA, USA). Additionally, a high-resolution transmission electron microscope (TEM; JEOL Ltd., Tokyo, Japan) fitted with an electron diffraction pattern was used to record transmission electron micrographs.

### 2.4. Animals and Experimental Design

Fifty female Swiss albino mice aged 6–8 weeks and weighing 16 to 23 g were obtained from the National Institute of Research (NIR), Giza, Egypt. The mice were provided a standard pellet diet and water ad libitum and maintained under standard conditions of temperature and humidity for one week for acclimation. Ehrlich solid carcinoma (ESC) cells obtained from NIR were examined and were found to be more than 99 percent viable by trypan blue dye exclusion.

On day 0, 0.2 mL of 2 × 10^6^ ESC tumor cells was inoculated subcutaneously into the left hind legs of the mice. The tumor-bearing mice were randomly assigned to five groups (n = 10) after five days. The ESC control group received only 1% Tween 80. Group 2: CPN-treated animals received a single dose of IP (5 mg CPN/kg) 10 days after the tumor cell injection [4]. Group 3: The VCN group received 40 mg VCN/kg/day orally [10]. Group 4: These animals received oral AgNPs [6 mg/kg/day; Rageh et al. [11]]. Group 5: The VCN-AgNP group consisted of mice that received oral VCN-AgNPs (20 mg/kg/day). Additionally, groups 1, 2, and 4 received 1 mL of 1% Tween 80/day orally.

On day 20 of the experiment, 7 mice from each group were anesthetized with ether before being executed through cervical dislocation. Tumor tissue was carefully removed, weighed, and divided into pieces. One portion was preserved in 10% formalin for histological investigation, while the remaining portions were frozen at −80 °C for future study. The remaining 3 mice from each group were used to calculate the MST.

### 2.5. Determination of Total Body Weight (BW), Tumor Weight (TW), and Tumor Volume

At the end of the 20th day, 7 mice from each group were weighed, then slaughtered, and the tumors were extracted and weighed in accordance with Rajkapoor et al. [12]. The tumor volume was assessed every two days with a Vernier caliper (Tricle, China). The volume of the developing tumor mass was determined using the following formula:

TV (mm^3^) = [(W^2^ × L)/2]

where W is the width of the minor tumor and L is the tumor length [13]. Furthermore, the tumor growth inhibition index (T/C%) was calculated based on the equation mentioned by Hather et al. [14] as follows:

T/C% = [1 − (mean volume of treated tumors)/(mean volume of control tumors)] × 100%.

### 2.6. Determination of the Median Survival Time (MST) and the Percentage Increase in the Median Life Span (%IMLS)

The formula to calculate MST was [first death + last death in the group]/2. Meanwhile, the formula for calculating %IMLS was [(MST in the treatment group/MST in the control group) − 1] × 100 [15].

### 2.7. Evaluation of Oxidant and Antioxidant Markers

Using kits obtained from Cayman Chemical Company (Ann Arbor, MI, USA), the reduced glutathione (GSH), lipid peroxidation (MDA), and nitric oxide (NO) levels in ESC lysate were evaluated using manufacturer-recommended methods. Furthermore, the activities of superoxide dismutase (SOD), catalase (CAT), glutathione reductase (GR), and glutathione peroxidase (GPx) in ESC homogenates were determined by the methods described by Sun et al. [16], Luck [17], Factor et al. [18], and Weydert and Cullen [19], respectively, by using commercially available kits. Meanwhile, the TAC Assay Kit (BioCat GmbH, Im Neuenheimer Feld, Heidelberg, Germany) was used to assess the levels of trolox equivalent antioxidant capacity (TAC) in the ESC homogenates, according to the manufacturer’s instructions.

### 2.8. Molecular Studies for the Evaluation of the Expression Rates of Bcl-2, Bax, and Casp-3 in ESC Tissues

The RNeasy Plus Minikit was used to purify the total RNA extracted from the ESC tissue. RevertAid H Minus reverse transcriptase was used to prepare the cDNA. The cDNA samples were subjected to real-time PCR testing. Applied Biosystems 7500 equipment was used to perform real-time PCR experiments using Power SYBR Green. The relative expression levels of the genes were adjusted for GAPDH.

The primer sequences of the genes are provided in Table 1.

### 2.9. Immunohistochemical Analysis (IHC)

The expression of the Bcl-2 and Bax proteins in the samples was assessed using immunohistochemical testing technology. Tumor tissue slices (4 μm) were treated overnight at 4 °C with rabbit anti-Bcl-2 and anti-Bax polyclonal antibodies (1:200, Abcam, Cambridge, UK). The samples were then stained with the avidin–biotin complex (ABC) as previously described by Ozer et al. [20]. Photographs were taken at 400× magnification with an ACCU-SCOPE 3000-LED microscope (New York Microscope Company, Hicksville, NY, USA). The positive reaction intensity of antibody staining was quantified using ImageJ software (version 1.54j, Java 1.1.4).

### 2.10. Determination of Tumor Necrosis Factor-α (TNF-α), Interleukin-1 Beta (IL-1β), and Vascular Endothelial Growth Factor (VEGF) in ESC Tissues by the ELISA Technique

The protein levels of the cytokines TNF-α, IL-1β, and VEGF were quantified in tumor homogenate using ELISA kits (Elabscience CO., Houston, TX, USA) for each cytokine, following the supplier’s guidelines.

### 2.11. Histopathological Examination

The tumor samples, which were 3–5 µm thick, were treated with a 4% paraformaldehyde solution to fix them. Following fixation, the samples were cut, dehydrated, embedded, sectioned, and stained with hematoxylin and eosin (H&E). Tumor tissues were examined using an Olympus BX 41 microscope (Japan). Necrosis, apoptosis, neovascularization, and inflammatory cell infiltration were semi-quantitatively assessed as follows: (−) none; (+) mild; (++) moderate; (+++) severe; (++++) more severe.

### 2.12. Statistical Analysis

Data are presented as the arithmetic mean ± standard deviation. Two groups were compared using Student’s *t*-test, and multiple groups were compared using one-way ANOVA and the Tukey test. Statistical significance was defined at *p* < 0.05. SPSS 20.0 and GraphPad Prism 8.0 performed the statistical analysis.

## 3. Results

### 3.1. Nanoparticle Characterization

**Figure 1:**
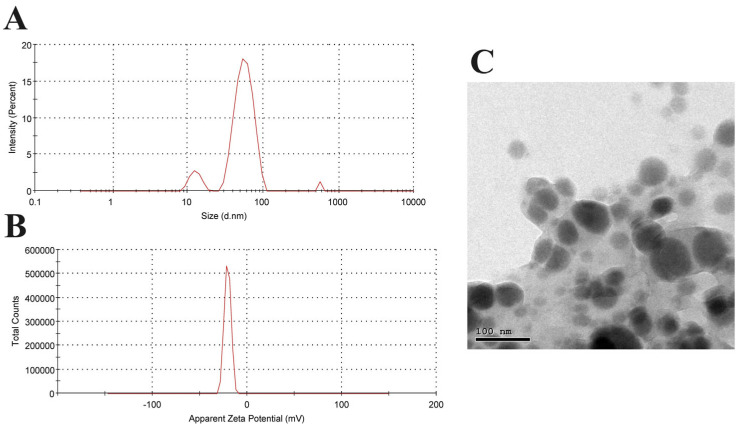
Characterization of VCN-AgNPs: (**A**) Hydrodynamic diameter of VCN-AgNPs by Zetasizer. (**B**) Surface charge of VCN-AgNPs determined by zeta potential. (**C**) Particle shape of VCN-AgNPs determined by transmission electron microscopy.

The synthesized VCN-AgNPs were characterized by an average diameter of 93.4 nm with a polydispersion index (PDI) of 0.437, as illustrated in Figure 1A. The NPs were smaller than 200 nm, which allowed them to easily evade the scavenging actions of monocytes and the reticuloendothelial system. As a result of the enhanced permeability and retention effect, which promotes greater internalization, they tended to accumulate at the tumor sites. The zeta potential was employed to analyze the surface charges of the AgNPs that were laden with VCN. The VCN-AgNPs were negatively charged and exhibited a value of −20.7 mV, which indicates that they were highly stable (Figure 1B). Additionally, the morphology of the biosynthesized VCN-AgNPs was examined by TEM. The VCN-AgNP TEM picture showed spherical particles with a diameter of less than 200 nm. These particles exhibited little to no agglomeration and were evenly distributed (Figure 1C).

### 3.2. Effect of VCN-AgNPs on Body Weight, Tumor Weight, Tumor Volume, and Tumor Growth Inhibition Index (T/C%)

**Figure 2:**
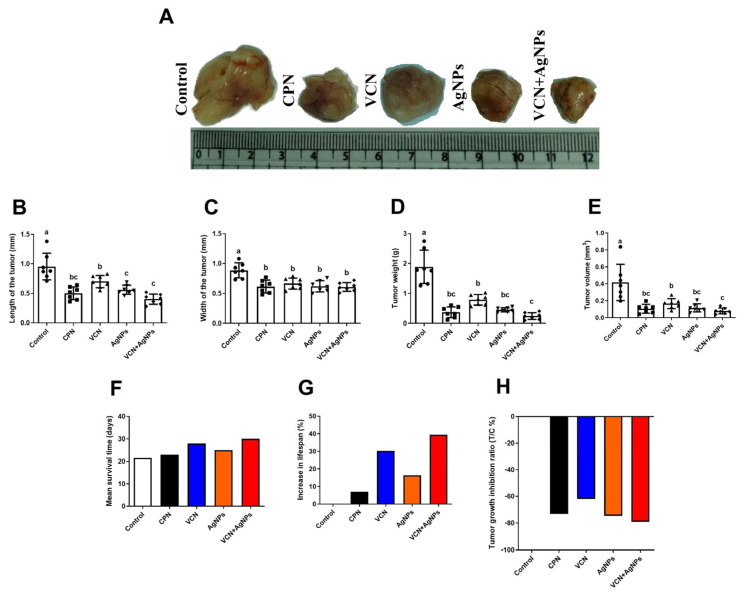
Effects of CPN, VCN, AgNP, and VCN-AgNP administration on tumor dimensions [(**A**) developed Ehrlich solid tumors (ESTs) in different groups, (**B**) length, (**C**) width], (**D**) tumor weight, (**E**) tumor volume, (**F**) mean survival time, (**G**) percentage IMLS, and (**H**) tumor growth inhibition index (T/C %) in Ehrlich solid carcinoma-bearing mice. The results are expressed as the mean + SD. Different letters show statistically significant differences between groups at *p* < 0.05.

As shown in Figure 2, the treatment of animals with VCN or VCN-AgNPs significantly (*p* < 0.05) reduced tumor growth, as indicated by the notable decrease in body weight and tumor width, length, and volume, as well as tumor weight, relative to the ESC group. Interestingly, no substantial differences in tumor weight or tumor volume were detected between the VCN-AgNP and CPN groups. Furthermore, the group receiving combination therapy (VCN-AgNPs) showed a more pronounced reduction in tumor growth (length, width, and volume). The tumor growth inhibition index is a widely used measure to estimate the effectiveness of treatments in drug screening investigations involving cancer xenografts. The T/C% index values for the CPN, VCN, AgNP, and VCN-AgNP groups were 72%, 61%, 73% and 77%, respectively, as shown in Figure 2. These findings demonstrated that, out of all the treatments under investigation, the combination of VCN-AgNPs produced the greatest T/C%.

### 3.3. Effect of VCN-AgNPs on MST and %IMLS

As shown in Figure 2, the MSTs in the ESC, CPN, VCN, AgNP, and VCN-AgNP groups were 21.5, 23, 28, 25, and 30 days, respectively. VCN alone or in combination with AgNPs prolonged the MST of the treated mice. Hence, treatment with VCN and VCN-AgNPs increased the percentage IMLS by 30.2% and 39.5%, respectively. Meanwhile, CPN and AgNPs increased the percentage IMLS by 6.9% and 16.3%, respectively. These results demonstrate the crucial role of VCN in improving the efficacy and safety of AgNPs, as the addition of VCN to AgNPs resulted in a 2.4-fold increase in %IMLS.

### 3.4. Effect of VCN-AgNPs on the Oxidant/Antioxidant Status

**Figure 3:**
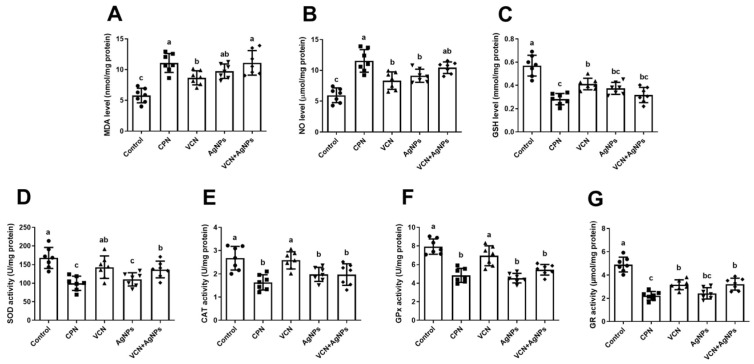
Effects of CPN, VCN, AgNP, and VCN-AgNP administration on oxidants [(**A**) malondialdehyde (MDA) and (**B**) nitric oxide (NO)] and antioxidants [(**C**) glutathione (GSH), (**D**) superoxide dismutase (SOD), (**E**) catalase (CAT), (**F**) glutathione peroxidase (GPx), and (**G**) glutathione reductase (GR)] in the ESC homogenates in Ehrlich solid carcinoma-bearing mice. The results are expressed as the mean + SD. Different letters show statistically significant differences between groups at *p* < 0.05.

As shown in Figure 3, there was a notable increase (*p* < 0.05) in oxidative stress marker (MDA and NO) levels, along with a substantial decline in antioxidant marker (GSH and TAC) levels in both the VCN and VCN-AgNP groups, compared to the ESC group. Although VCN alone did not exert a significant effect on antioxidant enzyme activity, the VCN-AgNP combination significantly decreased the activities of antioxidant enzymes (GR, CAT, SOD, and GPx) in contrast to the ESC group, indicating that the combination of VCN-AgNPs increased the oxidative effect. However, no notable differences were detected between VCN-AgNP- and CPN-treated animals in these oxidant/antioxidant parameters (MDA, NO, GSH, GPx, TAC, and CAT).

### 3.5. Effect of VCN-AgNPs on Apoptotic and Antiapoptotic Markers

**Figure 4:**
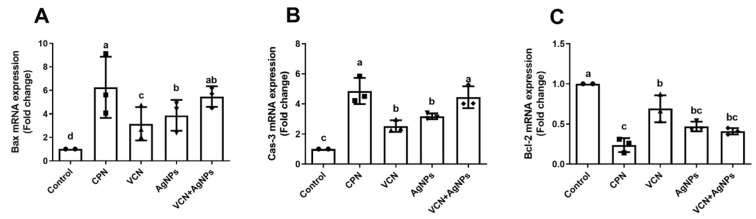
Effects of CPN, VCN, AgNP, and VCN-AgNP administration on mRNA expression of apoptotic proteins [(**A**) Bax, (**B**) Caspase -3, and (**C**) Bcl-2] in the ESC homogenates in Ehrlich solid carcinoma-bearing mice. The mRNA expression results are reported as the mean + SD of three experiments performed in duplicate and compared to GAPDH. Different letters mean statistically significant differences between groups at *p* < 0.05.

As shown in Figure 4, VCN and VCN-AgNPs dramatically increased the expression of genes for pro-apoptotic factors (*p* < 0.05), such as Bax and Casp-3, and considerably decreased the expression of the anti-apoptotic factor Bcl-2 gene compared to its relative expression in the ESC group without treatment (*p* < 0.05). Furthermore, there was no significant difference in the expression rate of the apoptosis parameters (Bax, Casp-3, and Bcl-2) between the VCN-AgNP and CPN groups.

### 3.6. Results of Immunohistochemical Examination

**Figure 5:**
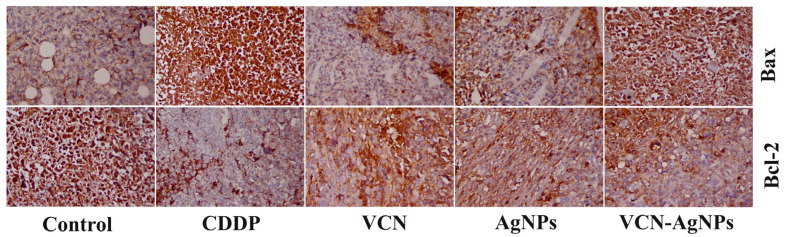
Immunostaining for Bax and Bcl-2 proteins in Ehrlich solid carcinoma tumor tissues in untreated mice and mice treated with CPN, VCN, AgNPs, and VCN-AgNPs. Magnification: 40×.

**Figure 6:**
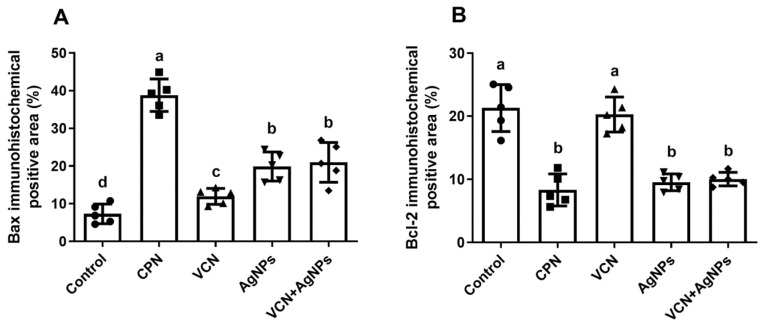
Effects of CPN, VCN, AgNP, and VCN-AgNP administration on (**A**) Bax immunohistochemical positive area (%) and (**B**) Bcl-2 immunohistochemical positive area (%) in the ESC tissue of Ehrlich solid carcinoma-bearing mice. Quantification of the area as a percentage was performed by ImageJ software; version 1.54j, Java 1.1.4. The results are expressed as the mean + SD of 5 fields. Different letters mean statistically significant differences between groups at *p* < 0.05.

To obtain insights into the mechanism of VCN or VCN-AgNPs in suppressing tumor growth, IHC investigations were used to assess changes in the expression and immunoreactivity of proteins associated with apoptosis in ESC tissues. Figure 5 and Figure 6 show that the animals treated with VCN and VCN-AgNPs had down-regulated Bcl-2 and up-regulated Bax protein expression. The observed results support our molecular studies and indicate that apoptosis induction may be a potential method by which VCN-AgNPs and VCN suppress tumor growth.

### 3.7. Effect of VCN-AgNPs on the Histology of ESC Tissues

**Figure 7:**
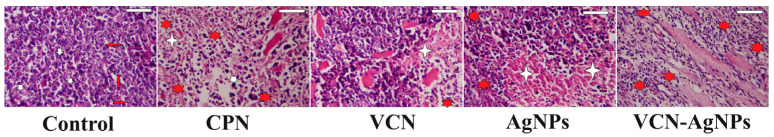
Histopathology of Ehrlich solid carcinoma tumors tissues in untreated mice and mice treated with CPN, VCN, AgNPs, and VCN-AgNPs (400×, scale bar = 100 μm). Apoptosis (red stars); necrosis (white stars); giant cells (short white arrows); and mitosis (red arrows). Magnification: 40×.

As shown in Figure 7, ESC cell implantation led to the development of solid tumors, manifested by a disturbance in tissue architecture and the presence of aggregated and condensed cancerous cells, along with significant cellular anaplasia, pleomorphism, and anisocytosis. This was accompanied by the appearance of highly chromatophilic tumor cells with nuclear vesicularity, atypicality, hyperchromasia, mitoses, and inconsistent morphology. Furthermore, neovascularization or the growth of new blood capillaries was observed, with little to no inflammatory response. In animals treated with CPN, severe necrosis and persistent cancer cells were observed encircling muscular tissue. It was shown that the ESC tissue continued to suffer damage after receiving VCN or AgNP therapy. The tumor appeared to have grown slowly and dispersed within these groupings, with a discontinuous appearance. Interestingly, mice receiving VCN-AgNPs showed a high regression of tumor development, wide and high zones of apoptotic cells, and many other zones of tumor cell remnants. A scoring of the histopathological findings is summarized in Table 2.

### 3.8. Effect of VCN-AgNPs on VEGF, IL-1β, and TNF-α

**Figure 8:**
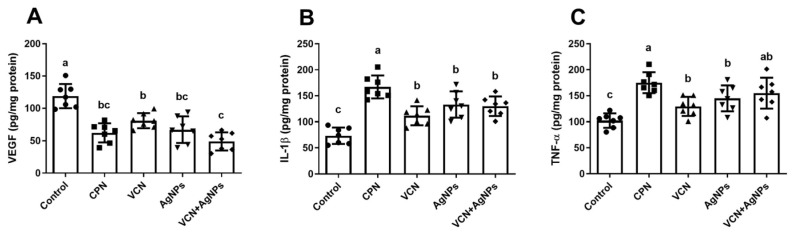
Effects of CPN, VCN, AgNP, and VCN-AgNP administration on (**A**) vascular endothelial growth factor (VEGF), (**B**) interleukin-1 beta (IL-1β), and (**C**) tumor necrosis factor-α (TNF-α) in the ESC homogenates in Ehrlich solid carcinoma-bearing mice. The results are expressed as the mean + SD. Different letters mean statistically significant differences between groups at *p* < 0.05.

Figure 8 shows a substantial reduction in the level of the powerful angiogenic factor VEGF in both the VCN group and the VCN-AgNP group compared to that in the ESC group (*p* < 0.05). Furthermore, the VEGF level in the VCN-AgNP group showed a considerable decrease compared to that in the VCN group (*p* < 0.05). However, no significant variations were observed in VEGF levels between the VCN-AgNP and CPN groups. The impact of VCN-AgNPs on the inflammatory cascade in malignant tissues was evaluated by evaluating the IL-1β and TNF-α levels. There was a notable increase in the inflammatory mediator (IL-1β and TNF-α) levels in the groups treated with VCN or VCN-AgNPs compared to the ESC group (*p* < 0.05). Furthermore, no notable disparities were observed in the values of these two parameters between the VCN-AgNP and CPN groups.

## 4. Discussion

Cancer remains a major health issue globally that requires the creation of new, safe medications. Continued research and innovation in the pharmaceutical sector are vital to improve treatment methods, reduce adverse effects, and improve patient prognosis. Prioritizing the development of effective and secure drugs is imperative in the fight against this pervasive illness [21].

The use of nanotechnology in combination therapy is now a highly persuasive approach for cancer treatment due to its superior effectiveness and reduced incidence of negative effects [7]. Therefore, this study aimed to evaluate the antitumor potential of VCN loaded in AgNPs in vivo using the ESC model, compared to the activity of a reference anticancer drug, cisplatin.

The results of this study showed that VCN-loaded AgNPs exerted a potent antiproliferative effect against ESC tumors in mice, as indicated by the marked decrease in body weight, tumor weight, and tumor size after VCN-AgNP administration. In addition, the combination of VCN and AgNPs was more effective in reducing tumor volume and weight than treatments with each drug individually. In particular, there were no discernible variations in ESC tumor size or weight between animals treated with CPN, a standard anticancer drug, and those treated with VCN-AgNPs. Interestingly, treatment with VCN-AgNPs resulted in the highest tumor growth inhibition index among all the groups, as shown in Figure 2.

The ability to prolong life is one of the most trustworthy metrics to assess the efficacy of any anticancer drug. Our findings showed that loading VCN onto AgNPs increased the percentage IMLS in mice by more than two times compared to AgNPs alone and more than five times compared to the CPN group. These findings further support and validate the efficacy and safety of VCN-AgNPs in the treatment of ESC.

This cytotoxic effect of VCN-AgNPs in ESC tissues was confirmed with a histological examination, where the administration of VCN-AgNPs led to a significant reduction in tumor progression and caused large areas of necrosis and cancer cell debris to appear. These findings are consistent with previous research supporting the cytotoxic effects of vincamine on cancer cells and its ability to regulate key proteins implicated in tumor development [22]. In particular, vincamine displays anticancer activity against many types of cancer cell lines, such as the lung cancer cell line A549 [23,24], the mouse melanoma B16 cell line [25], and human KB oral epidermoid carcinoma [26]. Similarly, Dhyani et al. [6] reported that VCN might attach to microtubulins, one of the important components of the cytoskeleton, to form the tubulin–VCN complex, which suppresses cancer cell migration and metastasis and causes cancer cell death. Furthermore, our findings align with a large body of evidence that demonstrates the capacity of AgNPs at various doses to break down tumors and reduce the weight and size of solid tumors [27].

Our results also support a prior report’s findings [28] that conjugating AgNPs with the natural alkaloid extract berberine increased their cytotoxic efficacy compared to the use of each agent alone. This improvement in effectiveness can be attributed to the enhanced permeability and retention effect, where tumor cells have a greater tendency to absorb nanoparticle-sized bodies compared to normal tissues. Furthermore, due to inadequate lymphatic drainage in the tumor, nanoparticles are able to remain and infiltrate. This may improve the specific drug delivery of VCN-AgNPs [29], given that the shape, size, and type of a nanoparticle influence its blood circulation, capacity to marginate, and rate of tumor deposition, as well as its therapeutic efficacy.

Because they trigger apoptosis and autophagy, ROS are being studied for their medicinal potential in cancer treatment. The cytotoxicity cascade begins with intracellular ROS production, which is a critical hallmark of early cellular responses to NPs. However, when NPs create enough intracellular ROS, cells stop functioning and die [30]. Corroborating this hypothesis, our findings recorded a significant elevation in oxidative stress in ESC homogenates from mice treated with VCN-AgNPs, as indicated by an elevation in ROS (MDA and NO) levels accompanied by a notable decrease in TAC, GSH, and antioxidant enzyme activities (GPx, GR, SOD, and CAT). These results indicated that the antitumor activity of VCN-AgNPs could be attributed in part to their pro-oxidant activity.

These findings were consistent with multiple studies that demonstrated that AgNPs can enter tumor cells, generate ROS, inhibit antioxidant molecules, and cause significant cellular injury, including mitochondrial and DNA damage, resulting in cell apoptosis or necrosis [11,31]. Furthermore, as shown in Figure 3, VCN alone increased oxidative stress in ESC tissues by altering the levels of MDA, NO, and GSH in tumor tissues. These results corroborate the assertions of recent studies that VCN induces oxidative stress in cancer cells through the augmentation of ROS production [23]. Meanwhile, Verma et al. [24] concluded that the level of ROS was increased after vincamine treatment, indicating ROS-mediated apoptosis in A549 cells. Thus, VCN synergizes with AgNPs and enhances oxidative stress in tumor tissues. 

Enhancing apoptosis to control tumor progression is the main mechanism pursued by most anticancer treatments [32]. Caspase-3 and Bax play a central role in apoptosis and are correlated with apoptosis rates in cancer. In the current study, molecular and immunohistochemical investigations confirmed that VCN and VCN-AgNP administration significantly enhanced the apoptotic cascade in tumor cells, as indicated by the up-regulation of the pro-apoptotic proteins Bax and Casp-3 and by the down-regulation of the anti-apoptotic protein Bcl-2 in ESC tissues. Furthermore, VCN-AgNPs were more efficient in inducing apoptotic markers than VCN or AgNPs alone, indicating the synergistic effect of VCN and AgNPs.

This apoptotic effect of VCN-AgNPs may be attributed to oxidative stress, as ROS can damage the mitochondrial membrane and release cytochrome c into the cytosol. This initiates the caspase signaling cascade, causing DNA damage. Furthermore, ROS can alter translocation, phosphorylation, and cleavage of the Bcl-2 protein, ultimately leading to apoptosis [33]. In support of our findings, Mavrogiannis et al. [34] reported that treatment of SKBR-3 breast cancer cells with vinca alkaloids increased the Bax/Bcl-2 mRNA ratio, indicating the creation of the intrinsic apoptotic pathway. Likewise, Dhyani et al. [6] reported that VCN had a dose-dependent effect on Casp-3 activity in A549 cells. Furthermore, pretreatment with a caspase inhibitor significantly reduced the cytotoxic effects of VCN in lung cancer cells. Therefore, it is clear that VCN-mediated caspase activation plays a crucial role in apoptosis [23,35]. Furthermore, many studies have shown that AgNPs alone can activate Casp-3 and endonuclease-activated DNase, which promote DNA fragmentation leading to apoptosis in breast cancer [32,36]. This elucidates the reason why the combination of AgNPs with vincamine enhances its apoptotic effect in cancerous tissues.

Additionally, the impact of VCN-AgNPs on the inflammatory cascade in malignant tissues was considered by evaluating the pro-inflammatory agents IL-1β and TNF-α, which have considerable pro-apoptotic effects in cells by increasing the expression ratio of Bax/Bcl-2, releasing cytochrome c from mitochondria, and driving Casp-9 and Casp-3 to achieve apoptosis [37,38].

In the current study, the administration of VCN or VCN-AgNPs significantly increased the levels of TNF-α and IL-1β in the ESC tissues compared to the ESC group. These findings suggest that the antitumor activity of VCN-AgNPs or VCN may be attributed to the induction of apoptosis via the activation of inflammatory mediators. The increase in inflammatory factors can be attributed to the oxidative impact of VCN and VCN-AgNPs, as ROS could activate NF-κB and p38 mitogen-activated protein kinase. This activation, in turn, leads to the creation of various inflammatory mediators, such as TNF-α and IL-1β [7]. Similar results were reported previously [37,39]. However, previous reports confirmed that the administration of AgNPs showed a notable increase in the levels of IL-1β and TNF-α and that these nanoparticles induced inflammatory immune responses [40,41].

Furthermore, interference in the angiogenesis pathway shows great potential in inhibiting the progression of cancer diseases. VEGF is certainly the most well-known trigger for angiogenesis. Our results revealed that the tumor content of VEGF was significantly decreased in animals treated with VCN or VCN-AgNPs compared to the ESC group. Moreover, VCN-AgNPs were more effective than VCN in reducing VEGF levels. These results indicate that the effectiveness of VCN-AgNPs in reducing tumor growth in ESC could be mediated in part by the inhibition of angiogenesis via a reduction in VEGF levels. In support of our findings, vinpocetine, a synthetic derivative of vincamine, prevented thioacetamide-induced liver fibrosis by inhibiting VEGF expression in the liver [42]. Furthermore, several studies showed that AgNPs alone could prevent angiogenesis by inhibiting VEGF by inactivating the Src kinase pathway [43]. Furthermore, AgNPs have been shown to inhibit VEGF in both dermal fibroblasts and dermal keratinocytes [44].

## 5. Conclusions

We can conclude that the coupling of vincamine with silver nanoparticles enhanced its therapeutic effectiveness and led to notable suppression of solid Ehrlich carcinoma in a mouse model. The combination of vincamine and silver nanoparticles can suppress Ehrlich solid carcinomas by inducing oxidative stress, inflammation, and apoptotic cascade, as well as by suppressing angiogenesis in tumor tissues. The mechanism of action of VCN-AgNPs could be connected to signaling pathways such as VEGF, Casp-3, Bax/Bcl-2, TNF-α, and IL-1β. In this regard, the current findings provide a unique concept of using VCN-AgNPs as a novel anticancer agent. Further studies of the anticancer mechanisms of VCN-AgNPs are needed to develop economical, reliable, and broad-spectrum anticancer agents.

## Figures and Tables

**Table 1:** Primer sequences of the *Bcl-2*, *Bax*, *Casp-3*, and *GAPDH* genes.

Genes	Forward Primer Sequence (5′->3′)	Reverse Primer Sequence (5′->3′)
GAPDH	AATGGGCAGCCGTTAGGAAA	GCGCCCAATACGACCAAATC
BCl-2	CCTATCTGGGCCACAAGTGAA	ACAGCCTGCAGCTTTGTTTC
BAX	CATGGGCTGGACATTGGACT	AAAGTAGGAGAGGAGGCCGT
Casp-3	GCGGATGGGTGCTATTGTGA	ACACAGCCACAGGTATGAGC

**Table 2:** Effects of CPN, VCN, AgNP, and VCN-AgNP administration on histopathology scoring of tumor-bearing mice.

	Necrosis	Apoptosis	Neovascularization	Inflammatory Cell Infiltration
Control	+	+	+++	+
CPN	++++	+++	+	++
VCN	+++	++	++	+
AgNPs	+++	+++	+	++
VCN+AgNPs	++++	++++	+	+++

(+) mild; (++) moderate; (+++) severe; (++++) more severe.

## Data Availability

All relevant data are within the paper.
